# Development and validation of age- and gender-specific reference intervals for the triglyceride-glucose index in a large Chinese healthy adult population

**DOI:** 10.3389/fendo.2026.1793587

**Published:** 2026-04-16

**Authors:** Shanshan Huang, Liming Qin, Chuanfu Lu, Weinan Li, Yibo Wang, Dan He, Yuni Mai, Na Lu, Qiuyu Lu, Shiwei Huang, Xiaowen Zheng, Jianfeng Zhang, Jihua Feng

**Affiliations:** 1 Department of Emergency Medicine, The Second Affiliated Hospital of Guangxi Medical University, Nanning, Guangxi, China; 2 Department of General Internal Medicine, Shibu Health Center, Nanning, Guangxi, China; 3 Department of Emergency Medicine, Wuming Hospital of Guangxi Medical University, Nanning, Guangxi, China

**Keywords:** triglyceride-glucose index, reference intervals, age, gender, Chinese population

## Abstract

**Background:**

The triglyceride-glucose (TyG) index is a recognized surrogate marker of insulin resistance. However, validated reference intervals (RIs) for the TyG index in large, general healthy populations are currently lacking, limiting its standardized application in clinical practice.

**Methods:**

This retrospective study established and validated TyG index RIs using data from adults (≥18 years) undergoing routine health examinations. The derivation cohort included individuals without known diabetes, dyslipidemia, or obesity. The TyG index was calculated as ln[fasting triglyceride (mg/dL) × fasting blood glucose (mg/dL)/2]. After outlier exclusion, we analyzed the age-TyG relationship using restricted cubic splines and threshold analysis to determine optimal age stratification. Gender- and age-specific RIs were defined as the 2.5th–97.5th percentiles. An independent cohort of 127,143 healthy individuals was used for validation, with success defined as <10% of values falling outside the proposed RIs.

**Results:**

A total of 201,623 individuals were initially screened for the derivation cohort. Analysis revealed a significant nonlinear relationship between TyG and age, with an inflection point at 64.21 years, justifying stratification into 18–64 and ≥64-year groups. The overall RI was 7.47–8.90. Stratified RIs were: 7.47–8.91 for males aged 18–64, 7.46–8.90 for females aged 18–64, 7.44–8.90 for males aged ≥64, and 7.50–8.90 for females aged ≥64. In the independent validation cohort, only 4.76% to 5.37% of values fell outside the corresponding RIs, confirming their robustness.

**Conclusion:**

This study establishes and validates age- and gender-stratified reference intervals for the TyG index in a large Chinese healthy population. These intervals, benchmarked against a critical age threshold of 64 years, provide a reliable standard for clinical interpretation and enhance the utility of the TyG index in metabolic risk assessment.

## Introduction

The triglyceride-glucose (TyG) index, derived from fasting triglyceride and glucose levels (1, 2), has emerged as a reliable and readily accessible surrogate marker of insulin resistance and systemic metabolic dysregulation (3, 4). Its core regulatory mechanisms can be categorized into the following three dimensions: synergistic network of insulin and related hormones, regulation of hepatic glucolipid output, uptake by skeletal muscle and adipose tissue (2, 5–8). Substantial evidence links elevated TyG index values to an increased risk of developing cardiovascular diseases, type 2 diabetes mellitus, and related metabolic disorders, highlighting its prognostic utility (9–11). However, despite its growing clinical relevance, standardized reference intervals (RIs) for the TyG index in general healthy populations remain inadequately defined. Current research focus has predominantly been on its predictive value and disease associations (12–14), rather than on establishing normative baselines.

Existing reference values are often extrapolated from studies involving specific patient cohorts or diseased populations, which may not accurately reflect the distribution within a healthy, community-dwelling demographic. Furthermore, the influence of key demographic factors, such as age and sex, on TyG index values is not well-characterized, and the potential necessity for stratified RIs has not been thoroughly investigated. A critical gap remains the lack of robust validation using large, independent cohorts to confirm the generalizability and clinical applicability of any proposed reference intervals.

To address these limitations, this study aimed to establish and validate clinically applicable reference intervals for the TyG index using a large retrospective dataset from a health examination population in Nanning, Guangxi.

The appropriate selection of statistical models during RIs establishment represents a critical determinant of both scientific validity and clinical applicability (15). Currently, international standards for reference interval establishment are predominantly framed around the Clinical and Laboratory Standards Institute (CLSI) EP28-A3c guideline, which employs parametric or nonparametric approaches to estimate reference limits encompassing the central 95% of the reference population (16). In recent years, the field has evolved toward greater refinement and individualization. For covariates such as age and sex, advanced statistical techniques including restricted cubic splines (RCS) and segmented regression analysis enable comprehensive characterization of the nonlinear relationships between covariates and biochemical parameters, thereby substantially expanding clinical utility (17–19). We performed a detailed analysis of the non-linear relationship between age and the TyG index, identified optimal age thresholds for stratification, and derived sex- and age-specific RIs. Subsequently, these intervals were rigorously validated in an independent cohort comprising over 120,000 individuals to ensure their robustness and reliability. The derivation cohort included 201,623 healthy individuals, and the independent validation cohort included 127,143 individuals. Such a large sample size far exceeds the minimum sample size requirement of at least 120 individuals for establishing reference intervals in the CLSI guideline, which can enhanced statistical power, reduced sampling error in percentile estimation, greater representativeness of the reference population, and the capacity for robust independent validation (20–22).

Our findings provide evidence-based, stratified reference intervals that may enhance the accurate clinical interpretation of the TyG index, thereby supporting its effective use in early metabolic risk assessment and preventive healthcare strategies.

## Methods

### Study population

A retrospective analysis was conducted using data from the Guangxi Primary Healthcare Information System (including multi-centers) at Shibu Health Center, Nanning, Guangxi. The study initially included adults (aged ≥18 years) who underwent routine health examinations between January 1 and December 31, 2024, and had results available for fasting triglycerides (TG) and fasting blood glucose (FBG). This study was approved by the Ethics Committee of the Second Affiliated Hospital of Guangxi Medical University [No. 2025-KYC(0560)].

**Figure 1:**
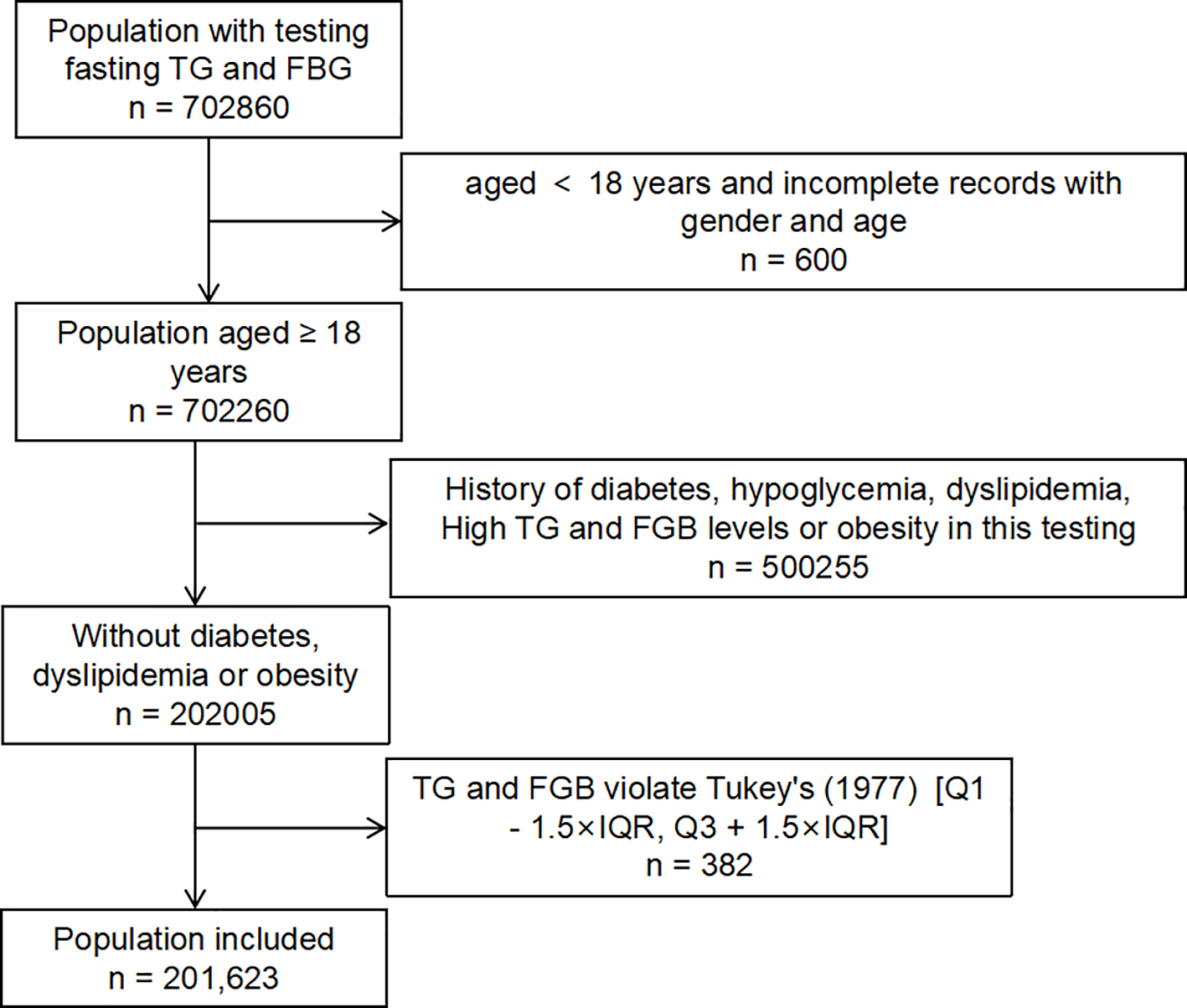
Flowchart of inclusion and exclusion of the study population for development of reference intervals of the TyG index.

Individuals with a known history of diabetes, dyslipidemia, or obesity [Body Mass Index, BMI ≥ 28 or waist circumference, WC ≥ 85 for female, WC ≥ 90 for male (23)] were excluded. Finally included participants were those aged ≥18 years with eligible blood samples. The detailed process of participant inclusion and exclusion is summarized in the flowchart (**Figure 1**). The inclusion and exclusion criteria were established in accordance with the CLSI EP28-A3c guideline for reference intervals, to ensure the validity of the derived reference ranges. The TyG index was calculated using the formula: TyG = ln [Fasting TG (mg/dL) × Fasting FBG (mg/dL)/2].

### Establishment and validation of reference intervals

The TyG index was calculated for all included participants using the aforementioned formula. To ensure robustness, potential outliers within the total population and each stratified subgroup were identified and excluded using Tukey’s (1977) method [Q1 - 1.5×IQR, Q3 + 1.5×IQR] (24). The reference intervals (RIs) were then defined as the 95% confidence intervals (P2.5–P97.5) for the overall population and for each subgroup (25).

For validation, a separate, retrospectively enrolled cohort of 127,143 ostensibly healthy individuals (aged ≥18 years) who underwent health examinations between January 1 and June 30, 2025, was used. The same inclusion and exclusion criteria applied to this validation cohort. The established RIs were considered valid and successfully established if the proportion of validation values falling outside the RIs was less than 10% (25).

### Statistical analysis

Data processing and statistical analysis were performed using R software (version 4.5.2) and the Zstats 1.0 online tool (www.zstats.net). Continuous data are presented as mean ± standard deviation or median (interquartile range, IQR), as appropriate, based on their distribution normality assessed by the Kolmogorov-Smirnov test. Group comparisons were made using the Chi-square test for categorical variables, the independent-sample t-test or Mann-Whitney U test for two-group comparisons of continuous variables, and the Kruskal-Wallis test followed by pairwise comparisons for multi-group analyses, where applicable. The relationship between age and the TyG index was analyzed using restricted cubic splines (RCS), and threshold analysis was employed to determine optimal age group boundaries. The RCS model was set with 4 knots placed at the 5th, 35th, 65th, and 95th percentiles of the age distribution, following Harrell’s recommendations for continuous variable modeling. The overall association and nonlinear component were each evaluated using the Wald chi-square test. For the threshold analysis, a segmented regression approach was used to identify the optimal breakpoint by minimizing the residual sum of squares across candidate thresholds; the significance of the threshold effect was evaluated by a likelihood ratio test comparing the segmented versus linear models. A two-sided P-value < 0.05 was considered statistically significant.

## Results

### General characteristics of main hematological parameters

As outlined in the flowchart (**Figure 1**), a total of 201,623 individuals were initially considered. Given that the TyG index was derived from FPG and TG levels, we excluded population whose FPG or TG values fell outside the Tukey (1977) outlier range [Q1 − 1.5 × IQR, Q3 + 1.5 × IQR] to ensure data reliability. The general characteristics of the included population, along with key hematological parameters stratified by gender and age, are summarized in **Tables 1**, **2**.

**Table 1:** General characteristics of TyG of 201,623 population based on age.

Variables	FPG (mg/dl), M (Q_1_, Q_3_)	TG (mg/dl), M (Q_1_, Q_3_)	TyG, M (Q_1_, Q_3_)
Total (n = 201623)	92.70 (83.70, 101.16)	89.43 (68.18, 113.34)	8.32 (8.03, 8.58)
18-30 years (n = 532)	87.21 (80.46,95.76)	77.92 (59.33,106.25)	8.15 (7.85,8.45)
30-40 years (n = 989)	89.10 (81.36,96.30)	81.46 (60.21,106.25)	8.19 (7.88,8.47)
40-50 years (n = 1457)	91.62 (83.16,100.44)	88.55 (66.41,113.34)	8.30 (8.00,8.57)
50-60 years (n = 3339)	92.70 (84.60,101.16)	91.20 (70.84,115.99)	8.35 (8.08,8.59)
60-70 years (n = 64775)	92.34 (83.88,100.80)	91.20 (69.95,115.11)	8.34 (8.05,8.59)
70-80 years (n = 89020)	92.70 (83.70,101.34)	89.43 (68.18,112.45)	8.32 (8.03,8.58)
80-90 years (n = 36382)	93.42 (83.70,102.42)	88.55 (67.29,110.68)	8.31 (8.02,8.56)
90-100 years (n = 4999)	93.60 (82.98,102.42)	88.55 (68.18,111.57)	8.31 (8.02,8.57)
≥100 years (n = 130)	90.72 (81.45,99.00)	88.10 (70.84,114.89)	8.30 (8.05,8.58)
P	<.001	<.001	<.001

**Table 2:** General characteristics of TyG of 201,623 population based on gender.

Variables	Male (n=94564)	Female (n=107059)	p.overall
Age (years), $\overset{—}{X}$ (SD)	73.2 (8.01)	73.6 (9.00)	<0.001
Age (years), n (%)			<0.001
18-30	220 (0.23%)	312 (0.29%)	
30-40	395 (0.42%)	594 (0.55%)	
40-50	611 (0.65%)	846 (0.79%)	
50-60	1181 (1.25%)	2158 (2.02%)	
60-70	31198 (33.0%)	33577 (31.4%)	
70-80	43681 (46.2%)	45339 (42.3%)	
80-90	15567 (16.5%)	20815 (19.4%)	
90-100	1687 (1.78%)	3312 (3.09%)	
≥100	24 (0.03%)	106 (0.10%)	
FPG (mg/dl)	92.9 [83.7;102]	92.3 [83.7;101]	<0.001
TG (mg/dl)	88.5 [66.4;112]	91.2 [70.0;114]	<0.001
TyG	8.30 [8.00;8.57]	8.34 [8.06;8.59]	<0.001

### RCS analysis and threshold analysis of TyG and age

**Figure 2:**
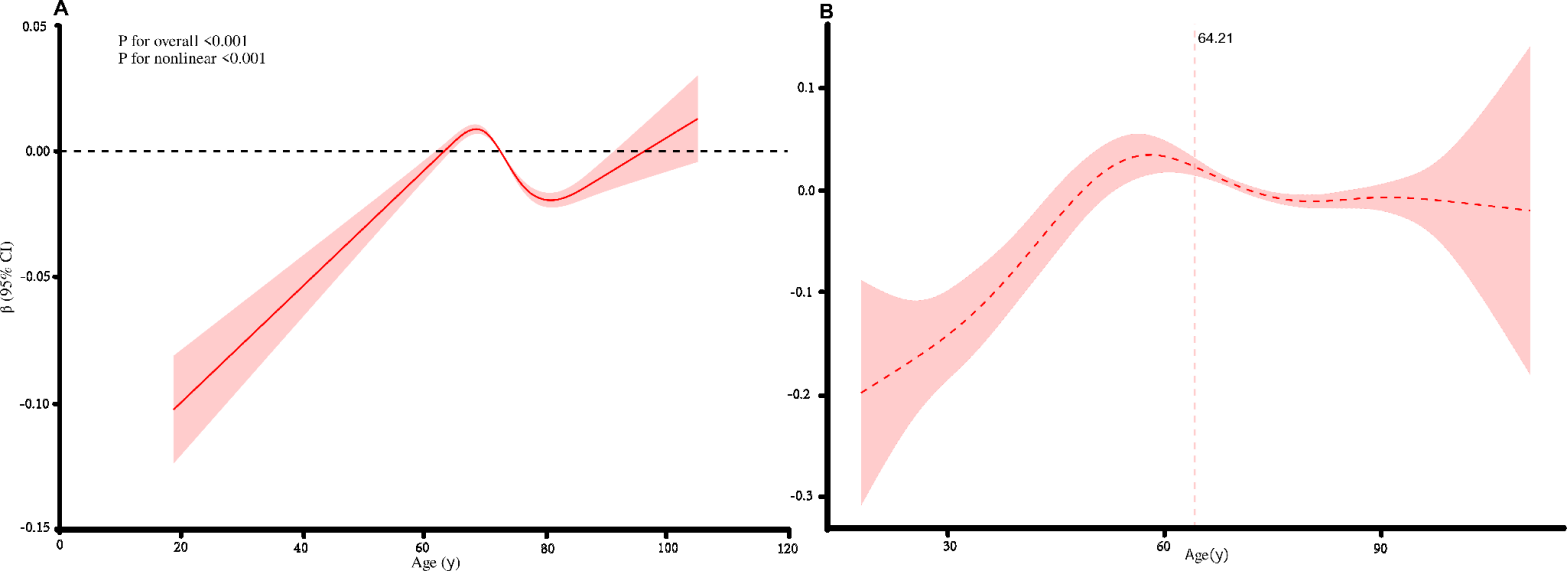
Nonlinear relationship between the TyG index and age, and the threshold analysis. **(A)** Association between the TyG index and age modeled using restricted cubic splines. A significant nonlinear relationship was observed (P for overall <0.001; P for nonlinear <0.001). **(B)** Segmented regression analysis identifying the threshold effect. A significant change in the slope of the TyG-age association was detected at the age of 64.21 years (P for the likelihood ratio test <0.001).

The association between TyG and age exhibited a nonlinear relationship (P for overall <0.001; P for nonlinear <0.001), as illustrated in **Figure 2A**. Threshold analysis was performed using a segmented regression model, which revealed a statistically significant threshold effect in the age- TyG association (P for likelihood ratio test <0.001). The corresponding results are presented in **Table 3** and visually detailed in **Figure 2B**.

**Table 3:** Threshold analysis for the age- TyG association.

Outcome	Effect	*P*
Model 1 Fitting model by standard linear regression	-0.00 (-0.00 - -0.00)	0.005
Model 2 Fitting model by two-piecewise linear regression		
Inflection point	64.21	
<64.21	0.01 (0.00 - 0.01)	<.001
≥64.21	-0.00 (-0.00 - -0.00)	<.001
P for likelihood test		<.001

### Establishment of reference intervals of TyG

Based on the aforementioned statistical results, it was necessary to stratify the reference intervals (RIs) for the TyG index by gender and age. The 95% confidence intervals (CIs; P2.5–P97.5) for the overall population were 7.47, 8.90. After removing outliers, RIs were calculated for each parameter and for each stratified subgroup, as summarized in **Table 4**.

**Table 4:** 95% confidence intervals (reference intervals) of TyG.

Items	Aged 18‐64 year		Aged ≥64 year	
	Male (n=3674)	Female (n=6097)	Male (n=90890)	Female (n=100962)
TyG (P2.5,P97.5)	7.47, 8.91	7.46, 8.90	7.44,8.90	7.50,8.90

### Validation of reference intervals of TyG

Based on Clinical and Laboratory Standards Institute (CLSI) document EP28-A3c—Defining, Establishing, and Verifying Reference Intervals in the Clinical Laboratory; Approved Guideline—Third Edition (25), we further validated the reference intervals (RIs) for TyG using an independent cohort of 127,143 healthy individuals. The proportion of validation values falling outside the established RIs was less than 10% (**Table 5**), indicating that the derived RIs are valid and appropriate for clinical use.

**Table 5:** Validations of reference intervals of TyG in a cohort of 127,143 healthy persons.

Items	Aged 18‐64 year		Aged ≥64 year	
	Male	Female	Male	Female
Sample number (n)	1996	3324	55393	66530
Number of below the down‐limit (n)	51	83	1525	1860
Number of above the upper‐limit (n)	44	82	1365	1715
Number of outside (n)	95	165	2890	3575
Outsider rate (%)	4.76%	5.12%	5.22%	5.37%

## Discussion

This study established and validated reference intervals (RIs) for the triglyceride-glucose (TyG) index in a large, ostensibly healthy Chinese population. The key findings are threefold. First, we identified a nonlinear relationship between age and the TyG index, with a significant inflection point at 64.21 years, indicating distinct age-dependent trends. Second, based on this threshold, we established gender- and age-stratified RIs for the TyG index, which exhibited minimal variation between genders within the same age group but showed a slight downward trend in the lower limit among older males. Third, these RIs were successfully validated in an independent, large-scale cohort, with less than 10% of values falling outside the proposed intervals, confirming their robustness and potential clinical utility.

The nonlinear association between TyG and age, characterized by an initial increase followed by a plateau or slight decline after approximately 64 years, aligns with and extends previous observations (26). Earlier studies often reported a linear positive correlation between TyG and age in younger or middle-aged populations (27). Our findings suggest that this relationship may not be linear across the entire adult lifespan. The identified inflection point near 64 years may reflect complex physiological shifts, including changes in body composition, hormonal levels (e.g., sex hormones), and lifestyle factors prevalent in older adulthood (28). This underscores the necessity of age stratification when establishing RIs for metabolic indices like TyG, as applying a single RI across all ages could lead to misinterpretation in older individuals.

A notable finding was the absence of substantial gender differences in TyG RIs within the same age stratum. While some studies report higher TyG values in males or postmenopausal women (29, 30), our results suggest that in a rigorously selected healthy population (excluding those with known dyslipidemia, diabetes, or obesity), the central 95% distribution of TyG is remarkably similar between genders. This implies that the same reference intervals may be applicable to both males and females for initial screening purposes, simplifying clinical interpretation. The slightly lower lower limit observed in males aged ≥64 years warrants further investigation but may relate to higher rates of subclinical metabolic changes or survival bias in this demographic (31).

The validation of the proposed RIs using a separate, contemporary cohort of over 120,000 individuals is a major strength of this study. Adhering to the Clinical and Laboratory Standards Institute (CLSI) document EP28-A3c—Defining, Establishing, and Verifying Reference Intervals in the Clinical Laboratory; Approved Guideline—Third Edition (16), the validation success rate (with out-of-range proportions consistently around 5%) strongly supports the generalizability and stability of our intervals. This external validation step is frequently omitted in similar studies but is critical for translating research findings into reliable clinical tools (32). Our RIs, therefore, provide a robust benchmark derived from and confirmed by large-scale real-world health examination data.

The core purpose of this study is to establish the physiological reference interval of TyG index in a large healthy Chinese population, which is used to define the normal range of TyG index in healthy people, and the statistical method is based on the percentile method recommended by CLSI guidelines. Previous studies on type 2 diabetes, chronic kidney disease and other populations mostly used logistic regression and ROC curve analysis to determine the disease diagnostic cut-off value of TyG index, which is used to distinguish the diseased population from the non-diseased population, and its core is to maximize the diagnostic efficiency, rather than define the normal physiological range (33–37). While logistic regression analysis dictates the initiation of clinical treatment, population-based RIs are essential for primary screening, allowing clinicians to differentiate baseline health from early subclinical deviations before overt disease manifests. The unprecedented scale of the current healthy cohort provides immense statistical power, enabling highly robust subpopulation partitioning and accurate outlier removal that simply cannot be achieved in smaller, disease-contaminated cohorts.

This study has several limitations. First, its cross-sectional design precludes establishing causal relationships between age and TyG trends, and does not allow assessment of how longitudinal changes in TyG values over time may affect correlations with other clinical parameters (38). Second, although we excluded individuals with known major metabolic diseases, residual confounding from undiagnosed conditions (such as non-alcoholic fatty liver disease or undiagnosed metabolic syndrome), the use of lipid/glucose-altering medications or unmeasured factors (e.g., diet, physical activity, alcohol consumption, and smoking status) cannot be entirely ruled out. Third, the population was drawn from a single geographic region in Southern China, which may limit direct extrapolation to other ethnic or geographic groups. Populations from different geographic regions may differ in dietary habits, lifestyle patterns, and genetic backgrounds, metabolic profiles vary substantially across ethnic groups, and even within China, regional metabolic heterogeneity exists. Furthermore, genetic factors may influence TyG distribution, particularly with respect to hereditary dyslipidemia or genetic variants in glucose metabolism pathways (39–42). Integrating genetic data into future reference interval studies would represent an important advance in the personalization of metabolic risk assessment. Future prospective, multi-center, multi-ethnic, longitudinal studies incorporating more detailed lifestyle and genetic data are needed to confirm and refine these intervals. Additionally, our study cohort was derived from individuals voluntarily undergoing routine health examinations. This population may be inherently more health-conscious and potentially healthier than the general public, introducing a potential “healthy volunteer bias.” Consequently, our RIs might be slightly narrower or lower than those derived from a truly unselected population sample, and caution should be exercised when extrapolating these findings to broader community settings.

## Conclusion

This large-scale study establishes and validates clinically applicable reference intervals for the TyG index, stratified by the critical age threshold of 64 years. These findings provide a much-needed standard for interpreting TyG values in the context of routine health assessments of Chinese adults. By confirming the nonlinear age relationship and offering validated RIs, our work facilitates the more accurate use of the TyG index as a simple, cost-effective tool for the early identification of individuals at potential metabolic risk, thereby aiding in targeted prevention strategies.

## Data Availability

The datasets presented in this article are not readily available because the data cannot be disclosed because it involves the privacy of the patients. If necessary, the original data can be obtained by emailing the corresponding author, but it cannot be disclosed. Requests to access the datasets should be directed to Jihua Feng, fengjihua@gxmu.edu.cn.
